# Risk conditions in children hospitalized with influenza in Norway, 2017–2019

**DOI:** 10.1186/s12879-020-05486-6

**Published:** 2020-10-19

**Authors:** Siri Helene Hauge, Inger Johanne Bakken, Birgitte Freiesleben de Blasio, Siri Eldevik Håberg

**Affiliations:** 1 Division of Infection Control and Environmental Health, Norwegian Institute of Public Health, Oslo, Norway; 2 Department of Health Registries, Norwegian Directorate of Health, Trondheim, Norway; 3 Department of Biostatistics, Oslo Centre for Biostatistics and Epidemiology, Institute of Basic Medical Sciences, University of Oslo, Oslo, Norway; 4 Norwegian Institute of Public Health, Centre for Fertility and Health, Oslo, Norway

**Keywords:** Influenza, Hospitalizations, Risk conditions, Children, Vaccination

## Abstract

**Background:**

Norwegian children are more frequently hospitalized with influenza than adults. Little is known about the characteristics of these children. Our aim was to investigate the presence of pre-existing risk conditions and to determine the duration of influenza hospitalizations in children during two influenza seasons.

**Methods:**

The Norwegian Patient Registry holds data on all hospitalized patients in Norway. We included all patients younger than 18 years hospitalized with a diagnosis of influenza during the influenza seasons 2017–18 and 2018–19. Pre-existing risk conditions for influenza were identified by ICD-10 diagnoses in the Norwegian Patient Registry. In addition, information on asthma diagnoses were also retrieved from the Norwegian Registry for Primary Health Care. To estimate the prevalence of risk conditions in the child population, we obtained diagnoses on all Norwegian children in a two-year period prior to each influenza season. We calculated age-specific rates for hospitalization and risk for being hospitalized with influenza in children with risk conditions.

**Results:**

In total, 1013 children were hospitalized with influenza during the two influenza seasons. Children younger than 6 months had the highest rate of hospitalization, accounting for 13.5% of all admissions (137 children). Hospitalization rates decreased with increasing age. Among children hospitalized with influenza, 25% had one or more pre-existing risk conditions for severe influenza, compared to 5% in the general population under 18 years. Having one or more risk conditions significantly increased the risk of hospitalization, (Odds Ratio (OR) 6.1, 95% confidence interval (CI) 5.0–7.4 in the 2017–18 season, and OR 6.8, 95% CI 5.4–8.4 in the 2018–19 season). Immunocompromised children and children with epilepsy had the highest risk of hospitalization with influenza, followed by children with heart disease and lung disease. The average length of stay in hospital were 4.6 days, and this did not differ with age.

**Conclusion:**

Children with pre-existing risk conditions for influenza had a higher risk of hospitalization for influenza. However, most children (75%) admitted to hospital with influenza in Norway during 2017–2019 did not have pre-existing risk conditions. Influenza vaccination should be promoted in particular for children with risk conditions and pregnant women to protect new-borns.

## Background

Influenza infection causes a substantial number of hospitalizations and deaths in young children each season [1–4]. Although accurate numbers of hospital admissions caused by influenza are difficult to establish, it has been estimated that among children under 5 years of age, annual hospitalizations world-wide are close to 15 million [5]. In Norway, with a population of approximately 5.3 million, it has been estimated that there are up to 5000 influenza-related hospitalizations yearly, with the highest rates in young children and the elderly [6].

Yearly vaccination is the most effective way to protect the high-risk population, and the World Health Organization recommends yearly influenza vaccination to those at risk for a severe outcome of influenza infection, including all children between 6 months to 5 years of age [7]. The primary goal for these recommendations is to lower the risk of severe influenza disease, complications and death. Some countries, like Finland and the United Kingdom, have implemented influenza vaccination in the childhood vaccination programs [8]. In Canada and the United States, influenza vaccination is recommended for the entire population but with emphasis on high-risk groups, including children between 6 months and 5 years of age [9, 10]. The current recommendations for yearly influenza vaccination in Norway include persons with the following risk conditions: chronic heart and lung disease, liver and/or kidney failure, chronic neurological disease or sequelae, BMI > 40, pregnant women, nursing home residents, and persons aged 65 and older [11]. Little is known about children hospitalized with influenza in Norway, and it is not known how hospitalizations are distributed among otherwise healthy children and children with risk conditions relevant for vaccination. Healthy children in Norway are not recommended influenza vaccination regularly.

Studies on risk conditions for severe influenza describe risk factors in adults, and conclusive studies on specific risk conditions are few, especially for the child population. Mertz et al. summarized risk conditions in adults and concluded that the presence of “any” risk conditions increased the risk of hospitalization and death; still the evidence for disease-specific risks were low [12]. Keren et al. concluded that neurological and neuromuscular disease were risk conditions for respiratory failure in children hospitalized with influenza [13]. A literature review by Havers et al. supported this finding, concluding that children with neurological conditions were overrepresented among patients hospitalized with influenza [14]. An Irish study by Rebolledo et al., concluded that children with risk conditions had an increased risk for severe influenza [15].

Better knowledge about underlying health conditions in young influenza patients can inform targeted vaccine recommendations, provide support for existing policies or guidance about new vaccine implementations, and help identify and prioritize communication towards targeted risk groups. We aimed to determine the characteristics and pre-existing conditions in patients younger than 18 years hospitalized with influenza during the two influenza seasons 2017–18, and 2018–19. Severity was also studied using length of hospitalization.

## Methods

### Study population and data sources

The study population included all residents in Norway under 18 years of age on January 1st 2018 (for evaluation of the 2017–18 influenza season), or on January 1st 2019 (for the 2018–19 season). Total population numbers were obtained from the official website of Statistics Norway [16]. This study was based on statistical data from Norwegian registries. Data are defined as statistics when all combinations of values for demographic and diagnostic groups appear for five or more individuals. Release of statistical data from the registries does not require ethical approval. We retrieved statistics from two national Norwegian health registries: 1) the Norwegian Patient Registry (NPR) and 2) The Norwegian Registry for Primary Health Care (NRPHC) [17]. NPR holds data from all hospitals in Norway [18]. The NPR holds information such as personal identification number, age, dates for hospitalization and discharge and diagnoses classified by the International Classification of Diseases (ICD-10). The NRPHC includes data from all contacts in primary care. This registry holds reimbursement data from primary care contacts including the patient’s personal identification number, date of consultation and diagnoses classified by the International Classification of Primary Care (ICPC-2).

### Influenza hospitalizations

We retrieved data on all hospitalized patients below 18 years of age diagnosed with influenza as defined by any of the ICD-10 codes J09-J11 (influenza) from October 2017 (week 40) to May 2018 (week 20) and from October 2018 (week 40) to May 2019 (week 20). These periods cover the surveillance periods for two influenza seasons in Norway and coincides with the standard surveillance periods for the European Centre for Disease Control (ECDC) [19]. We also retrieved data on length of stay.

### Risk conditions for severe influenza

We obtained the number of children in Norway within each age group (0–4 years, 5–9 years, 10–14 years and 15–17 years) by risk condition. Risk conditions were defined by registry information for 2 years prior to the start of each influenza season. These risk conditions were: Heart disease, lung disease, kidney disease, liver disease, neurological disease or sequela, diabetes 1 and 2, compromised immune system (including cancer patients and patients with transplants) and some birth defects and chromosomal disorders (categorized as “other conditions”). We received similar information for children hospitalized with influenza. Obesity (Body Mass Index > 40) was not included as a risk condition in our study due to the low number of registrations. As asthma is primarily treated in primary care, and we obtained diagnoses of asthma from the Norwegian Registry for Primary Health Care in addition to the NPR. Two registrations of asthma with the ICPC-2 code R96 were required to be defined as having asthma. Asthma (diagnosed in primary care) was analysed separately and also combined with lung conditions diagnosed in specialist care, and this group was defined as “any lung condition”. See the Additional file 1 for the diagnostic codes (ICD-10 and ICPC-2) used to categorize risk groups. Children not registered with any of these codes during the 2 years prior to each season were defined as having no pre-existing risk condition. The group defined as having “any risk condition” included all children with any of the diagnoses listed in Additional file 1. We received additional data on the number of children hospitalized with influenza by 1 year age groups, and for the age group 0–6 months separately.

### Statistical analyses

Rates for hospitalization were calculated for 1-year age groups, and in addition for children in the age group 0–6 months. Prevalence estimates on the defined risk conditions in the general population were calculated as the number of children diagnosed with risk conditions divided by the total population number in each age group: 0–4 years, 5–9 years, 10–14 years and 15–17 years. We compared the prevalence of risk conditions in influenza-hospitalized children with the prevalence in the general population. Odds ratios were obtained by first estimating the odds of influenza-hospitalization within a risk groups (e.g. number of influenza-hospitalized with a risk conditions divided by the number of children with a risk conditions who were not influenza-hospitalized). We divided this number with the odds of influenza-hospitalization among children who were not in a risk group (number of influenza-hospitalized without a risk conditions divided by those without a risk condition who were not influenza-hospitalized). The duration of hospital stay for influenza was calculated in days for all children hospitalized with influenza.

## Results

**Fig. 1:**
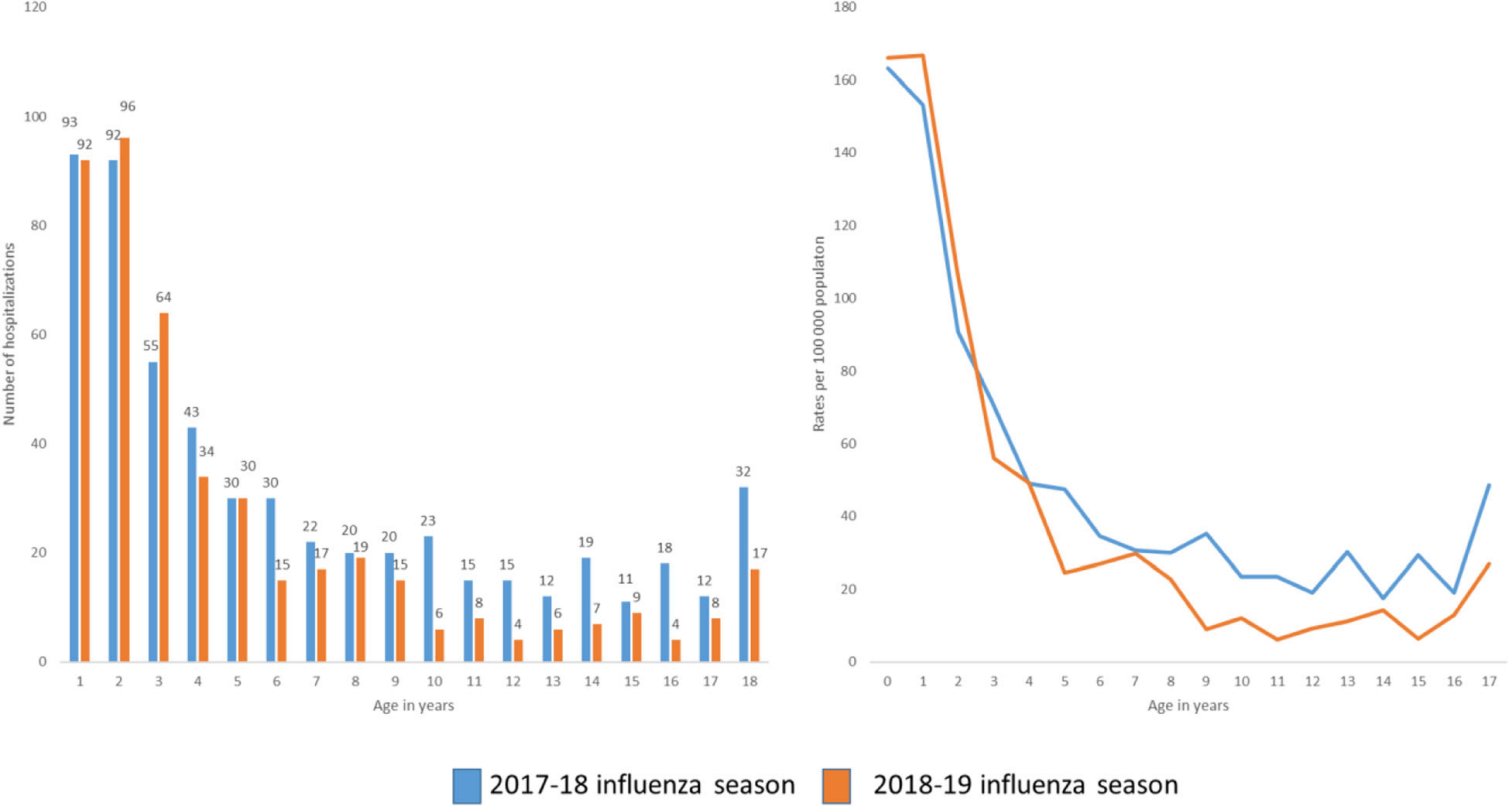
**a** and **b** Numbers and rates of influenza hospitalizations in children below 18 years, 2017–19, Norway

**Fig. 2:**
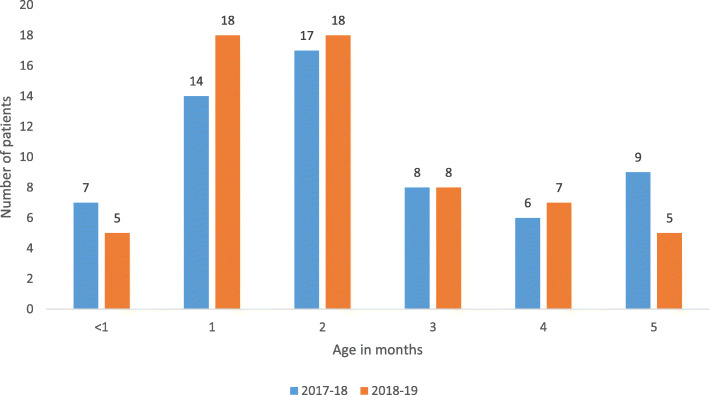
Number of children below 6 months of age hospitalized with influenza during the 2017–18 and 2018–19 influenza season, Norway

During the two influenza seasons, 2017–18 and 2018–19, a total of 1013 children were hospitalized with influenza in Norway, 562 in the 2017–18 influenza season, and 451 in the 2018–19 season. Twelve children were registered with influenza hospitalizations in both seasons. The highest numbers and rates of hospitalizations were found in the youngest children (Figs. 1a, b and 2), with those between 0 and 6 months of age showing the highest hospitalization rates (239 and 242 per 100,000 in 2017–18 and 2018–19, respectively), see Additional files 2 and 4. These represented 13.5% of all children hospitalized with influenza in the two seasons combined. For those younger than 1 year and those 1 year of age, 93 and 92 children were hospitalized with influenza in the 2017–18 influenza season (corresponding to 163 and 153 per 100,000 population). For the 2018–19 season, there were 92 and 96 hospitalizations among those younger than 1 year and those 1 year of age, respectively, corresponding to 166 and 167 per 100,000 population. Hospitalization rates declined with increasing age (Fig. 1, Additional file 2). There were less than five in-hospital deaths related to influenza in both seasons combined.

Among all children in Norway, 5.0–5.1% were registered with one or more of the risk conditions for severe influenza (Table 1).

**Table 1:** Number of children with risk conditions among children hospitalized with influenza and in the general child population

Risk group	Proportion (number) of hospitalized influenza patients with risk condition		Proportion (number) of children in the general population with risk condition	
	2017–18 season	2018–19 season	2017–18 season Population: 1129007	2018–19 season Population: 1122508
Lung disease^a^	12.1% (68/562)	13.5% (61/451)	3.4% (37,980/1129007)	3.3% (36,618/1122508)
Asthma^b^	6.1% (34/562)	4.9% (22/451)	2.2% (24,615/1129007)	2.1% (23,466/1122508)
Heart conditions	2.1% (12/562)	–	0.3% (3272/1129007)	0.3% (3295/1122508)
Immunocompromised	5.5% (31/562)	4.2% (19/451)	0.3% (3617/1129007)	0.3% (3630/1122508)
Neurological disease^c^	7.5% (42/562)	5.3% (24/451)	0.8% (8967/1129007)	0.8% (8995/1122508)
Epilepsy	5.7% (32/562)	4.4% (20/451)	0.5% (5643/1129007)	0.5% (5664/1122508)
Diabetes Mellitus 1	–	–	0.3% (2980/1129007)	0.3% (3052/1122508)
Any risk condition^d^	24.6% (138/562)	26.2% (118/451)	5.1% (57,237/1129007)	5.0% (56,125/1122508)

^a^Including asthma diagnoses in primary care and other lung conditions from specialist care

^b^Asthma diagnoses from primary care registry only

^c^Including epilepsy

^d^Including all diagnostic groups, also groups with numbers < 5 in individual groups

**Fig. 3:**
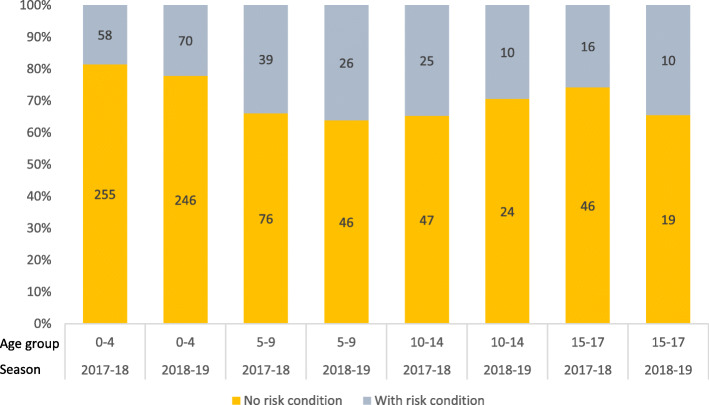
Proportion (%) (y-axis) and number (within bars) of children hospitalized with influenza who were in a risk group or not in any risk group, based on pre-defined risk conditions for severe influenza. Norway 2017–19

Lung disease (including asthma) was the most prevalent risk condition. In the total population, 3.4% (2017–18) and 3.3% (2018–19) were diagnosed with a lung condition. The second most common risk condition was neurological disease (Table 1 and Additional file 3). Among children hospitalized with influenza, 24.6–26.2% were categorized as having at least one risk condition (Table 1). The proportion of hospitalized children with a risk condition ranged from 18.5% in children 0–4 years in the 2017–18 season to 36.1% in children 5–9 years (Fig. 3). Having at least one risk condition was associated with an increased risk of hospitalization with influenza (OR 6.1, 95% CI 5.0–7.4 in the 2018–17 season, and OR 6.8 95% CI 5.4–8.4 in the 2018–19 season) when compared to children with no risk conditions (Table 2).

**Table 2:** Odds ratios with 95% confidence intervals, for being hospitalized with influenza in children with a risk condition during the influenza seasons in 2017–18 and 2018–19 in Norway

Risk category	Age groups (years)				
	0–4	5–9	10–14	15–17	All ages
	OR (95% CI)	OR (95% CI)	OR (95% CI)	OR (95% CI)	OR (95% CI)
**Influenza season 2017–18**					
Lung disease	3.0 (2.1–4.2)	4.5 (2.4–7.8)	2.6 (0.8–6.3)	5.8 (2.4–12.3)	4.0 (3.0–5.1)
Asthma	2.4 (1.5–3.7)	2.7 (1.0–6.0)	n.a.	6.3 (2.2–14.7)	2.9 (2.0–4.1)
Heart conditions	6.2 (2.0–14.7)	30.6 (12.0–65.6)	n.a.	n.a.	7.6 (3.9–13.3)
Neurological disease or sequelae	5.8 (2.5–11.5)	11.8 (5.5–22.5)	26.7 (14.0–47.8)	16.9 (7.3–34.6)	10.1 (7.2–13.9)
Epilepsy	6.8 (2.2–16.1)	16.2 (7.2–32.0)	25.1 (11.5–49.4)	23.4 (9.6–49.6)	12.1 (8.2–17.3)
Immunocompromised	11.4 (5.6–20.7)	44.5 (22.8–79.9)	35.1 (13.5–76.7)	n.a.	18.3 (12.3–26.4)
All patients with one or more risk condition					**6.1 (5.0–7.4)**
**Influenza season 2018–19**					
Lung disease	4.1 (3.0–5.6)	4.4. (1.9–9.0)	n.a.	n.a.	4.7 (3.5–6.1)
Asthma	2.5 (1.6–3.9)	n.a.	n.a.	n.a.	2.4 (1.5–3.7)
Heart conditions	n.a.	n.a.	n.a.	n.a.	n.a.
Neurological disease or sequelae	11.0 (6.1–18.6)	17.6 (7.7–35.7)	n.a.	n.a.	7.0 (4.4–10.5)
Epilepsy	16.9 (8.6–30.1)	23.8 (9.8–49.9)	n.a.	n.a.	9.2 (5.6–14.4)
Immunocompromised	8.9 (4.0–17.1)	26.1 (8.2–64.1)	n.a.	53.9 (16.0–144.6)	13.6 (8.1–21.6)
All patients with one or more risk condition					**6.8 (5.4–8.4)**

n.a. = not applicable due to low numbers

Patients diagnosed as immunocompromised were registered in 4.2% (in 2017–18) and 5.5% (in 2018–19) of the children hospitalised with influenza, compared to 0.3 in the general population. Being immunocompromised caused the highest risk for hospitalization with influenza (OR 18.3, 95% 12.3–26.4 in the 2018–17 season, and OR 13.6, 95% CI 8.1–21.6 in the 2018–19 season).

Epilepsy was registered in 5.3% (2017–18 season) and 4.4% (2018–19 season) of children hospitalized with influenza, compared to 0.5% in the general population. Hence, for epilepsy patients, there was an increased risk of hospitalization with influenza, OR in 2017–18 of 12.1 (95% CI 8.2–17.3) and OR in 2018–19 of 9.2 (95% CI 5.6–14.4) (Tables 1 and 2).

Heart conditions was registered in 2.1% of the children hospitalized with influenza, compared to 0.3% in the general population. We observed an elevated risk of hospitalization with influenza among these children (OR 7.6, 95% CI 3.9–13.3 in 2017–18). Of the children hospitalized with influenza, 12.1–13.5% had a lung disease, making this the most common risk condition among children hospitalized with influenza. Lung disease was registered in 3.3–3.4% of the general child population. Both asthma (diagnosed in primary care) (OR 2.9, 95% CI 2.0–4.1 in 2017–18 and OR 2.4, 95% CI 1.5–3.7 in 2018–19) and any lung disease (combined data from primary care on asthma and other lung conditions in specialist care) were associated with a slightly increased risk of being hospitalized with influenza (OR 4.0, 95% CI 3.0–5.1 in 2017–18, OR 4.7, 95% CI 3.5–6.1 in 2018–19).

There were less than five children hospitalized with influenza, who in addition was registered in NPR with one of the following diagnoses: diabetes I and II, kidney disease, liver disease, obesity and “other conditions”.

The 1013 patients hospitalized with influenza had a total of 4701 days in hospitals during the two seasons (2456 days in the 2017–18 season, and 2245 days in the 2018–19 season), with an average of 4.6 days per hospitalization (Additional file 2). Children aged 0–6 months were hospitalized for a total of 647 days during the two seasons. The average number of days of hospitalization did not differ between children older and younger than 6 months (4.6 vs 4.7, respectively).

## Discussion

During the two 2017–18 and 2018–19 influenza seasons, more than 1000 children under the age of 18 years were hospitalized with influenza in Norway. The majority (75%) of these children were not registered with pre-existing risk conditions for severe influenza. Still, children hospitalized with influenza had more often pre-existing risk conditions compared to the general population within the same age group.

Children under the age of 2 years, and especially those under 6 months of age, were more frequently hospitalized with influenza than older children. Our finding is in line with observations from the United Kingdom [20]. Asthma and other lung diseases were the most common risk conditions, both in the general population and among children hospitalized with influenza, and were associated with increased risk for hospitalization. However, more rare risk conditions, such as “compromised immune system” and “epilepsy” were also associated with substantially increased risks of being hospitalized.

We found that 5% of the total Norwegian child population were registered with one or more risk conditions, which is in line with estimates from other studies. A Swedish report, which also used hospital data to calculate the prevalence of risk groups in the population, found that approximately 7.5% of children had one or more risk condition, and a study from the United Kingdom estimated that 3.2% of children aged 0–14 years had a risk condition [21, 22]. Higher numbers have been found in a US study, in which it was estimated that 4–12% of children below 18 had one or more risk condition [23], while another study with self-reported data found that 7–14% of all children had a risk condition for severe influenza infection [24].

Previous studies have shown substantial variations in the proportion of children hospitalized with influenza who had pre-existing risk conditions. Estimates range from 12.9% in Austria, 35% in Ireland and 43% in both Bavaria, Germany and Philadelphia, US [13, 15, 25–29]. During the influenza A(H1N1) pandemic in 2009, it was estimated that 31% of hospitalized patients globally (among all ages), had one or more risk condition [30]. Children have a lower prevalence of chronic diseases than a combined prevalence estimate for all ages, as several established risk conditions, such as chronic heart and lung conditions are much more common among older patients.

One explanation for the relatively high proportion of hospitalized children with no pre-existing risk condition could be that there is a low threshold for hospitalization of children with influenza in Norway. Children, and in particular the youngest ones, are at high risk of complications from lung infections [31]. We observed that the youngest children below 6 months had higher hospitalizations rates than those older than 6 months. Dehydration is a possible complication of influenza and children, in particular new-borns, are more difficult to rehydrate and observe clinically than adults. Because of this, the threshold to hospitalize children is lower than for adults. We found that the average length of hospitalization for influenza for all children (4.6 days) was somewhat shorter than previously found for all ages in Norway (5.4 days) [6]. For children below 6 months the average length of stay was even shorter, with 4.0 days. This suggests that the hospitalized children does not require more hospital care than adults and that the children are not more severe sick than adults, and supports the assumption that the threshold for admission to hospitals for children may be lower than for adults.

Another reason why we found a high proportion of children with no pre-existing risk condition could be that children with risk conditions receive adequate treatment for their primary condition, which in turn, could lower their risk for severe influenza and admission to hospital.

Lung condition was the most frequently registered risk condition in our study, and we found an increased risk of hospitalization in children with a lung condition. Associations, although not strong, between asthma and severe outcome of influenza infection have also been found by others [12, 30], but not all. McLean et al. did not find that asthma increased the risk of severe illness in children [32]. It is difficult to obtain accurate prevalence estimates of asthma in children, as diagnostic criteria and data sources vary between countries. A Norwegian study based on data from the national prescription database found that 1 in 20 children aged 0–19 years (5%) had used anti-asthmatic drugs for more than 3 months [33], which is higher than our estimate based on primary care consultation data. However, as influenza can cause worsening of asthma symptoms, some patients may be registered with asthma diagnosis and not an influenza-diagnosis when hospitalized with influenza [34]. If so, we have missed some influenza-hospitalizations in this group of children, which may have attenuated our associations between asthma and influenza. This could also be the case for other conditions that may be exacerbated by influenza.

We found that 0.5% of the children in the total population were registered with epilepsy, in agreement with findings from Finland and UK [35, 36]. Our results indicate that epilepsy was associated with a substantially higher risk for hospitalization with influenza. Epilepsy has previously been identified as a risk factor for severe influenza infections and death in children [13, 37], supporting our finding of epilepsy as a risk condition for severe influenza. Influenza vaccination is recommended for children with epilepsy due to the well documented increased risk of seizures caused by viral infections, causing fever [38, 39]. Our results clearly show an increased risk of influenza hospitalization for children with heart conditions or who were immunocompromised. This finding supports the current recommendation that children with such conditions should be vaccinated. Both immunosuppression and neurological disease have previously been linked with an increased risk of death among patients with influenza infection [40], and Coffin et al. found that neurological disease and cardiac disease caused prolonged hospital stay in those younger than 21 years of age [41].

Due to the low numbers of children identified with diabetes mellitus in our study, we could not evaluate the risk in this group. A more extensive study is needed to be able to obtain risk estimates for this patient group.

Influenza vaccines are not approved for patients younger than 6 months [42]. The finding that children under 6 months are overrepresented among hospitalized children provides a strong argument for vaccinating pregnant women. Vaccination of pregnant women will not only protect the mother from influenza complications more often seen in pregnant and post-partum women [12, 30] but also protect the infant the first months after birth by transfer of maternal antibodies to the foetus.

When using registry data to identify risk conditions, there are several limitations. The sensitivity for capturing risk conditions depends on whether all diagnoses on existing conditions are registered during health care visits, and that patients regularly see a doctor for their condition. A Norwegian study in adults found that only 60% of adult patients with diabetes had seen a specialist the last year [43]. However, children in Norway with chronic conditions such as diabetes or epilepsy, are very likely to have annual visits and check-ups in specialist care and are thereby expected to be correctly defined in this registry-based study. Our prevalence estimates of the selected risk conditions are in line with other studies, supporting that we have identified most children with known risk conditions in Norway.

The susceptibility of influenza infection in children varies with the type of influenza virus. Surveillance reports show that the 2017–18 season in Norway was dominated by the influenza B Yamagata virus, and the 2018–19 season was dominated by the influenza A(H1N1) virus [44, 45]. Influenza B viruses have been seen to cause a higher number of severe infections and fatalities in children compared to influenza A outbreaks [37, 46]. This could be reflected in our findings of a slightly higher number of hospitalizations in the 2017–18 season. We did not have information on laboratory confirmation on influenza as the hospital registry does not provide information on laboratory tests. However, some of the ICD-10 codes for influenza do require laboratory confirmation, and tests are increasingly used in Norway when patients are hospitalized with influenza [47]. We therefore believe that the risk of misclassifying influenza in hospitalized patients is relatively low. Also, we excluded out-of-season diagnoses to reduce further the probability of including patients with other causes of infection wrongly classified as influenza. Moore et al. found a sensitivity of using ICD-10 codes to identify influenza hospitalizations of 86% and a very high specificity of 98% [48]. It is unlikely that all children hospitalized with influenza have been tested for influenza, which can cause underreporting of the disease. Also, the children could be registered with diagnoses due to complications of their primary condition and not including influenza as additional diagnose. Thus, the number of influenza-attributable hospitalizations in our study could be underestimated.

Another limitation of this study is the lack of information on the vaccination coverage as influenza vaccination will influence the hospitalization rate. However, children in Norway are not routinely recommended seasonal influenza vaccination, and in general, seasonal influenza vaccination rates are low in children. Children with risk conditions are recommended annual influenza vaccinations. Although the vaccination rate is considered to be insufficient [45], vaccination of high-risk children has probably prevented some hospitalizations. For this reason, children in risk groups may have an even higher underlying risk of severe influenza and hospitalization.

## Conclusion

Younger children were more often hospitalized with influenza than older children. Children with pre-existing risk conditions had a higher risk of being hospitalized with influenza than children without risk conditions. Still, most children hospitalized for influenza did not have pre-existing risk conditions. Our findings support existing vaccine recommendations for high-risk children. Recommendations of influenza vaccination to all children above 6 months of age could be considered. Our results strongly support the vaccination of pregnant women to protect the infants in the first few months of life.

## Supplementary information

**Additional file 1.** ICD-10 and ICPC codes used to classify children according to risk group.

**Additional file 2.** Numbers of children, rates and duration of hospitalizations in Norway, 2017–18 and 2018–19, by age.

**Additional file 3.** Number of children with different risk conditions among children hospitalized with influenza, and the numbers with these conditions in the general population, in 2017–19, Norway. Numbers 0 > < 5 are indicated as *.

**Additional file 4.** Number of children and rates of hospitalization in children younger than 6 months of age in Norway during the 2017–18 and 2018–19 influenza seasons.

## Data Availability

The datasets used and/or analysed during the current study are available from the corresponding author on reasonable request. The datasets generated and/or analysed during the current study are also available on application to the NPR repository, https://www.helsedirektoratet.no/tema/statistikk-registre-og-rapporter/helsedata-og-helseregistre/norsk-pasientregister-npr/sok-om-data-fra-npr.

## Abbreviations

CI: Confidence Interval
ECDC: European Centre for Disease Prevention and Control
ICD: International Classification of Disease
ICPC: International Classification of Primary Care
NPR: Norwegian Patient Registry
NRPHC: Norwegian Registry for Primary Health Care
OR: Odds Ratio
