# Correction to: Risk conditions in children hospitalized with influenza in Norway, 2017–2019

**DOI:** 10.1186/s12879-021-05913-2

**Published:** 2021-02-22

**Authors:** Siri Helene Hauge, Inger Johanne Bakken, Birgitte Freiesleben de Blasio, Siri Eldevik Håberg

**Affiliations:** 1 Division of Infection Control and Environmental Health, Norwegian Institute of Public Health, Oslo, Norway; 2 Department of Health Registries, Norwegian Directorate of Health, Trondheim, Norway; 3 Department of Biostatistics, Oslo Centre for Biostatistics and Epidemiology, Institute of Basic Medical Sciences, University of Oslo, Oslo, Norway; 4 Norwegian Institute of Public Health, Centre for Fertility and Health, Oslo, Norway

**Correction to: BMC Infect Dis 20, 769 (2020)**

**https://doi.org/10.1186/s12879-020-05486-6**

Following publication of the original article [1], an error was identified in one author’s affiliations: Dr. Siri Helene is also affiliated to the Department of Biostatistics, Oslo Centre for Biostatistics and Epidemiology, Institute of Basic Medical Sciences, University of Oslo, Oslo, Norway.
