# Supplementary material for: Risk conditions in children hospitalized with influenza in Norway, 2017–2019
Source: BMC Infect Dis. 2020 Oct 19;20:769. doi: 10.1186/s12879-020-05486-6 (PMC7569759; doi:10.1186/s12879-020-05486-6)
Supplement: Supplementary file 1 — Additional file 1. ICD-10 and ICPC codes used to classify children according to risk group. [file 12879_2020_5486_MOESM1_ESM.docx]

Additional file 1.

Classification (ICD-10 and ICPC codes*) used to define risk groups for severe influenza

| Risk group | ICD-10 codes (specialist care) |
| --- | --- |
| Diabetes 1 | E10, 024.0 |
| Diabetes 2 | E11, E12, E13, E14, G59.0, G63.2, N08.3, O24.1, P70.2, |
| Chronic lung disease (including asthma) | J4, J6, J7, J82, J84, J98.0-J98.6, Q30-34, Z99.0, Z99.1, E84. |
| Chronic heart conditions | I05-09, I11-15, I20-25, I27, I28, I31, I34-39, I42-45, I47-51, Z99.4. |
| Liver failure | K70-74 |
| Kidney failure | N07, N08.3, N18-19. Z99.2 |
| Chronic neurological disease or sequelae | G10-14, G20-26, G30-32, G35-37, G40-41, G70-73, G80-83, I60-69, Q02-03, Q05, Q06 |
| Immunosuppression** | B20-24, D56-58, D60-64, D70-71, D72.0, D72.8, D73.0, Q89.0, D74, D80-84, D86, D89, C (all codes), Z21, Z51.0, Z51.1, Z85, Z94. |
| Other severe or chronic disease*** | E24, Q90, Q91 |
| Risk group | **ICPC-2 code (primary care)** |
| Asthma | R96 |

* Codes with 2 digits include all diagnoses that begin with these two digits.
** Includes malignancies
***Includes syndromic disorders and Cushings syndrome
