# Supplementary material for: Risk conditions in children hospitalized with influenza in Norway, 2017–2019
Source: BMC Infect Dis. 2020 Oct 19;20:769. doi: 10.1186/s12879-020-05486-6 (PMC7569759; doi:10.1186/s12879-020-05486-6)
Supplement: Supplementary file 2 — Additional file 2. Numbers of children, rates and duration of hospitalizations in Norway, 2017–18 and 2018–19, by age. [file 12879_2020_5486_MOESM2_ESM.docx]

Additional file 2. Table: Number of hospitalizations, rates and duration of hospitalizations in Norway, 2017-18 and 2018-19, by age.

| Season | Age in years | Number of hospitalizations | Rates per 100 000 population | Total number of hospitalization-days | Average days per hospital stay |
| --- | --- | --- | --- | --- | --- |
| 2017-18 | 0 | 93 | 163 | 450 | 4,8 |
|  | 1 | 92 | 153 | 555 | 6,0 |
|  | 2 | 55 | 91 | 262 | 4,8 |
|  | 3 | 43 | 71 | 138 | 3,2 |
|  | 4 | 30 | 49 | 114 | 3,8 |
|  | 5 | 30 | 48 | 92 | 3,1 |
|  | 6 | 22 | 35 | 106 | 4,8 |
|  | 7 | 20 | 31 | 57 | 2,9 |
|  | 8 | 20 | 30 | 179 | 9,0 |
|  | 9 | 23 | 35 | 59 | 2,6 |
|  | 10 | 15 | 24 | 46 | 3,1 |
|  | 11 | 15 | 23 | 43 | 2,9 |
|  | 12 | 12 | 19 | 36 | 3,0 |
|  | 13 | 19 | 30 | 52 | 2,7 |
|  | 14 | 11 | 18 | 64 | 5,8 |
|  | 15 | 18 | 29 | 68 | 3,8 |
|  | 16 | 12 | 19 | 52 | 4,3 |
|  | 17 | 32 | 49 | 83 | 2,6 |
| Total |  | **562** | **50** | **2456** | **4.4** |
| 2018-19 | 0 | 92 | 166 | 406 | 4,4 |
|  | 1 | 96 | 167 | 403 | 4,2 |
|  | 2 | 64 | 106 | 389 | 6,1 |
|  | 3 | 34 | 56 | 207 | 6,1 |
|  | 4 | 30 | 49 | 131 | 4,4 |
|  | 5 | 15 | 24 | 103 | 6,9 |
|  | 6 | 17 | 27 | 37 | 2,2 |
|  | 7 | 19 | 30 | 95 | 5,0 |
|  | 8 | 15 | 23 | 187 | 12,5 |
|  | 9 | 6 | 9 | 27 | 4,5 |
|  | 10 | 8 | 12 | 35 | 4,4 |
|  | 11 | 4 | 6 | 14 | 3,5 |
|  | 12 | 6 | 9 | 6 | 1,0 |
|  | 13 | 7 | 11 | 24 | 3,4 |
|  | 14 | 9 | 14 | 59 | 6,6 |
|  | 15 | 4 | 6 | 30 | 7,5 |
|  | 16 | 8 | 13 | 23 | 2,9 |
|  | 17 | 17 | 27 | 69 | 4,1 |
| Total |  | **451** | **40** | **2245** | **4.9** |
