# Supplementary material for: Risk conditions in children hospitalized with influenza in Norway, 2017–2019
Source: BMC Infect Dis. 2020 Oct 19;20:769. doi: 10.1186/s12879-020-05486-6 (PMC7569759; doi:10.1186/s12879-020-05486-6)
Supplement: Supplementary file 3 — Additional file 3. Number of children with different risk conditions among children hospitalized with influenza, and the numbers with these conditions in the general population, in 2017–19, Norway. Numbers 0 > < 5 are indicated as *. [file 12879_2020_5486_MOESM3_ESM.docx]

Additional file 3.Number of children with different risk conditions among children hospitalized with influenza, and the numbers with these conditions in the general population, in 2017-19, Norway. Numbers 0> <5 are indicated as *.

| Season 2017-18 | | | | | | | | | | | | | | | | | | | |
| --- | --- | --- | --- | --- | --- | --- | --- | --- | --- | --- | --- | --- | --- | --- | --- | --- | --- | --- | --- |
|  | Age groups (years) | | | | | | | | | | | | | | | | | | |
|  | 0-4 | | | 5-9 | | | | 10-14 | | | | | 15-17 | | | | All ages | | |
| Total child population | 299579 | | | 323672 | | | | 315683 | | | | | 190073 | | | | 1129007 | | |
| Hospitalized with influenza diagnose | 313 | | | 115 | | | | 72 | | | | | 62 | | | | 562 | | |
|  | **Hospitalized** | **In total population** | | **Hospitalized** | | **In total population** | | **Hospitalized** | | | **In total population** | | **Hospitalized** | | **In total population** | | **Hospitalized** | | **In total population** |
| Lung disease | 40 | 14086 | | 15 | | 10452 | | 5 | | | 8706 | | 8 | | 4736 | | 68 | | 37980 |
| Asthma | 22 | 9100 | | 6 | | 6601 | | * | | | 5746 | | 6 | | 3168 | | 34 | | 24615 |
| Heart conditions | 5 | 787 | | 7 | | 691 | | * | | | 886 | | * | | 908 | | 12 | | 3272 |
| Neurological disease or sequelae | 8 | 1359 | | 10 | | 2612 | | 15 | | | 3097 | | 9 | | 1899 | | 42 | | 8967 |
| Epilepsy | 5 | 718 | | 9 | | 1697 | | 10 | | | 2024 | | 8 | | 1204 | | 32 | | 5643 |
| Immunocompromised | 11 | 969 | | 13 | | 937 | | 7 | | | 973 | | * | | 738 | | 31 | | 3617 |
| Season 2018-19 | | | | | | | | | | | | | | | | | | | |
|  | Age groups (years) | | | | | | | | | | | | | | | | | | |
|  | 0-4 | | | | 5-9 | | | | 10-14 | | | 15-17 | | | | All ages | | | |
| Total child population | 294863 | | | | 320053 | | | | 320042 | | | 187550 | | | | 1122508 | | | |
| Hospitalized with influenza diagnose | 316 | | | | 72 | | | | 34 | | | 29 | | | | 451 | | | |
|  | **Hospitalized** | | **In total population** | | **Hospitalized** | | **In total population** | | **Hospitalized** | **In total population** | | **Hospitalized** | | **In total population** | | **Hospitalized** | | **In total population** | |
| Lung disease | 52 | | 13445 | | 9 | | 9985 | | * | 8558 | | * | | 4630 | | 61 | | 36618 | |
| Asthma | 22 | | 8450 | | * | | 6265 | | 0 | 5642 | | * | | 3109 | | 22 | | 23466 | |
| Heart conditions | * | | 807 | | * | | 689 | | * | 893 | | * | | 906 | | - | | 3295 | |
| Neurological disease or sequelae | 15 | | 1340 | | 9 | | 2582 | | * | 3211 | | * | | 1862 | | 24 | | 8995 | |
| Epilepsy | 12 | | 701 | | 8 | | 1680 | | * | 2098 | | * | | 1185 | | 20 | | 5664 | |
| Immunocompromised | 9 | | 982 | | 5 | | 919 | | * | 1002 | | 5 | | 727 | | 19 | | 3630 | |
