# Supplementary material for: Risk conditions in children hospitalized with influenza in Norway, 2017–2019
Source: BMC Infect Dis. 2020 Oct 19;20:769. doi: 10.1186/s12879-020-05486-6 (PMC7569759; doi:10.1186/s12879-020-05486-6)
Supplement: Supplementary file 4 — Additional file 4. Number of children and rates of hospitalization in children younger than 6 months of age in Norway during the 2017–18 and 2018–19 influenza seasons. [file 12879_2020_5486_MOESM4_ESM.docx]

Additional file 4.

Table: Number of children and rates of hospitalization in children younger than 6 months of age in Norway during the 2017-18 and 2018-19 influenza seasons.

| Season | Age in months | Number of hospitalizations | Total  duration of hospitalizations | Mean duration of hospitalizations | Population* | Rate per  100 000 population |
| --- | --- | --- | --- | --- | --- | --- |
| 2017-18 | <1 | 7 | 16 | 2,3 | 4747 | 147 |
|  | 1 | 14 | 119 | 8,5 | 4747 | 295 |
|  | 2 | 17 | 48 | 2,8 | 4747 | 358 |
|  | 3 | 8 | 63 | 7,9 | 4747 | 169 |
|  | 4 | 6 | 18 | 3,0 | 4747 | 126 |
|  | 5 | 9 | 55 | 6,1 | 4747 | 190 |
|  |  |  |  |  |  |  |
| 2018-19 | <1 | 5 | 15 | 3,0 | 4616 | 108 |
|  | 1 | 18 | 41 | 2,3 | 4616 | 390 |
|  | 2 | 18 | 47 | 2,6 | 4616 | 390 |
|  | 3 | 8 | 20 | 2,5 | 4616 | 173 |
|  | 4 | 7 | 42 | 6,0 | 4616 | 152 |
|  | 5 | 5 | 9 | 1,8 | 4616 | 108 |
| Total |  | **122** | **493** | **4,0** |  |  |
| *The population of 0-year olds divided by 12 was used as proxy as we did not have access to population figures by birth month | | | | | | |
